# Primary Mucinous Adenocarcinoma of the Renal Pelvis: A Case Report

**DOI:** 10.7759/cureus.62709

**Published:** 2024-06-19

**Authors:** Mary V Nueva Espana-Perez, David G Pedroza

**Affiliations:** 1 Pathology and Laboratory Medicine, Corazon Locsin Montelibano Memorial Regional Hospital, Bacolod, PHL

**Keywords:** primary mucinous adenocarcinoma of the renal pelvis, mucinous adenocarcinoma, pseudomyxoma, myxoma, adenoma carcinoma sequence, kidney myxoma, pseudomyxoma nephrii

## Abstract

Mucinous adenocarcinomas of the upper urinary tract are extremely rare, and the majority of information available comes from case reports or short case series. Intestinal metaplasia is considered a premalignant condition in carcinogenesis.

Here we present a case of a 55-year-old male who presented with a left flank mass extending to the left hemiabdomen and macroscopic hematuria. Pathologic findings revealed that the kidney was transformed into a multiloculated cystic mass measuring 18 × 12 × 11 cm, with a dilated pelvicalyceal system obstructed by a stone at the renal hilus and filled with a gray, soft, gelatinous material. Histopathologic sections showed glandular metaplasia of the proximal ureter ascending to the renal pelvis, lined by intestinal-type columnar epithelium containing goblet cells admixed with malignant glands floating in abundant extracellular mucin, along with poorly differentiated areas composed of signet ring cells.

Immunohistochemistry studies showed positive periodic acid Schiff (PAS)/periodic acid Schiff-diastase (PAS-D), consistent with the mucinous nature of the intracellular and extracellular materials. Positive immunohistochemical staining for CK7, CK20, and CDX2 (CK7+/CK20+/CDX2+) highlighted the intestinal differentiation of this neoplasm. This offers evidence for the intestinal metaplasia-dysplasia-carcinoma sequence rather than a teratomatous or coelomic epithelial origin in mucinous ovarian-like cystadenocarcinoma involving the renal pelvis.

## Introduction

A mucinous carcinoma of the renal pelvis is a rare neoplasm, with intestinal metaplasia of the transitional epithelium thought to be the major morphologic precursor [1-4]. In this case study, we present a 55-year-old male with a long history of chronic flank pain with hematuria and a palpable abdominal mass. Post-operatively, the kidney was found to be cystic and filled with mucin, as a result of the primary malignant neoplasm displaying signet ring cell features and extensive mucin production. A carcinogenic sequence of intestinal metaplasia-dysplasia-carcinoma in situ was observed in the proximal ureter. Here we define this sequence through the use of standard histochemical and immunohistochemical studies, supporting intestinal metaplasia as a precursor lesion for the rare adenocarcinoma of the renal pelvis.

## Case presentation

A 55-year-old male presented with a mass on the left flank extending to the left hemiabdomen, which was gradually increasing in size. The mass was associated with pain, hematuria, and pyuria. Ultrasonography revealed the complex nature of the mass and confirmed hypernephroma, while CT imaging confirmed hydronephrosis secondary to a ureteropelvic junction calculus. Several complex indeterminate hepatic nodules measuring 0.5 to 2 cm in widest diameter were observed.

**Figure 1:**
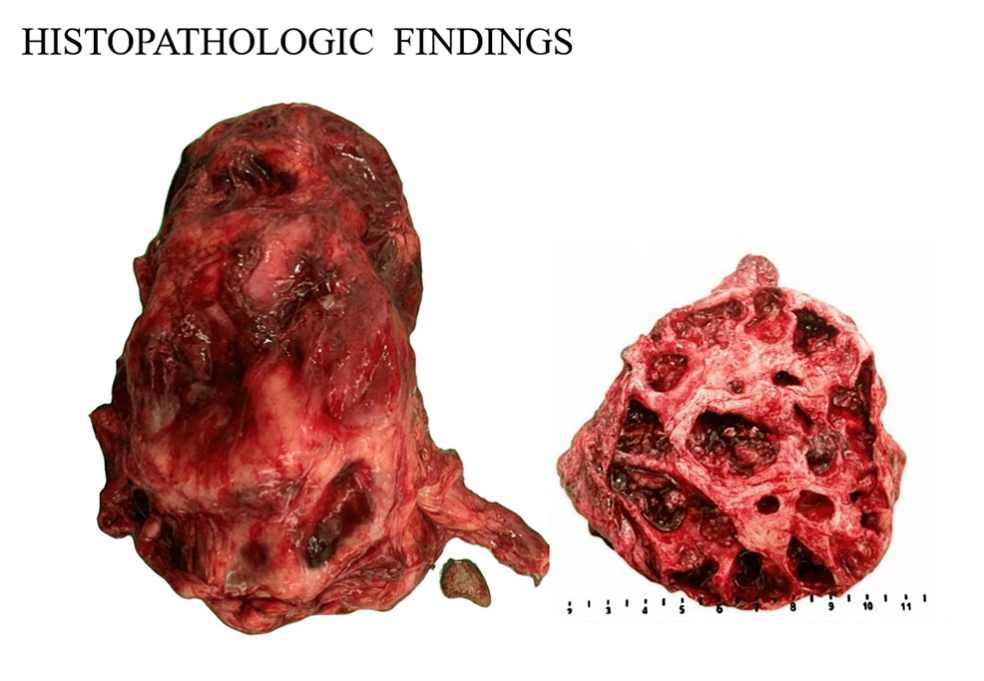
The kidney in a 55-year-old male was transformed into a multiloculated cyst with a renal stone obstructing the renal hilus. The cut section reveals the dilated pelvicalyceal system and cystic cavities filled with mucin and necrotic debris.

**Figure 2:**
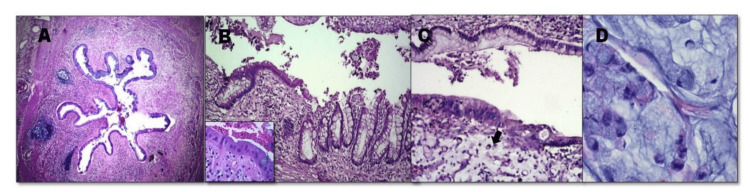
(A) Ureter showing intestinal metaplasia (HE 40X). (B) The transition zone transitional-intestinal metaplasia-dysplasia (inset) sequence with increasing nuclear irregularities (HE 100X). (C) Dysplastic epithelium with an underlying poorly differentiated carcinoma (arrow) (HE 100X). (D) Signet ring cells make up the poorly differentiated areas and are seen floating in mucin (HE 400X).

The kidney was transformed into a multiloculated cyst with a markedly dilated pelvicalyceal system and a renal stone measuring 3 × 2 × 1.4 cm obstructing the renal hilus. The cystic cavities and dilated pelvis were filled with gray-white mucin and necrotic debris (Figure 1). Histologic sections from the pelvis revealed a malignant epithelium composed of multilayered haphazardly arranged tall columnar epithelial cells associated with abundant mucin. Stromal and renal parenchymal invasions were noted, with signet ring cell changes seen floating in lakes of mucin. Microscopic sections from the ureter revealed a transitional epithelium gaining gradual metaplastic changes, which transforms into varying degrees of dysplasia and subsequently advances into a frank carcinoma (Figure 2).

Histochemistry and immunohistochemistry

Periodic Acid-Schiff (PAS) With Diastase

Tissues from the renal pelvis and kidney parenchyma were fixed in 10% neutral buffered formalin, paraffin-embedded, and then cut into 4-μm-thick sections. Histochemical staining was performed using the Merck PAS staining kit (Merck KGaA, Darmstadt, Germany). Two identical sections were taken from the renal pelvis, deparaffinized, and rehydrated. The first slide was treated with saliva at 37˚C for 15 min, serving as the a-amylase with proper controls performed. The second slide was rehydrated only and then treated, together with the first slide, with periodic acid solution and Schiff’s reagent, and counterstained with Modified Gill’s Hematoxylin.

**Figure 3:**
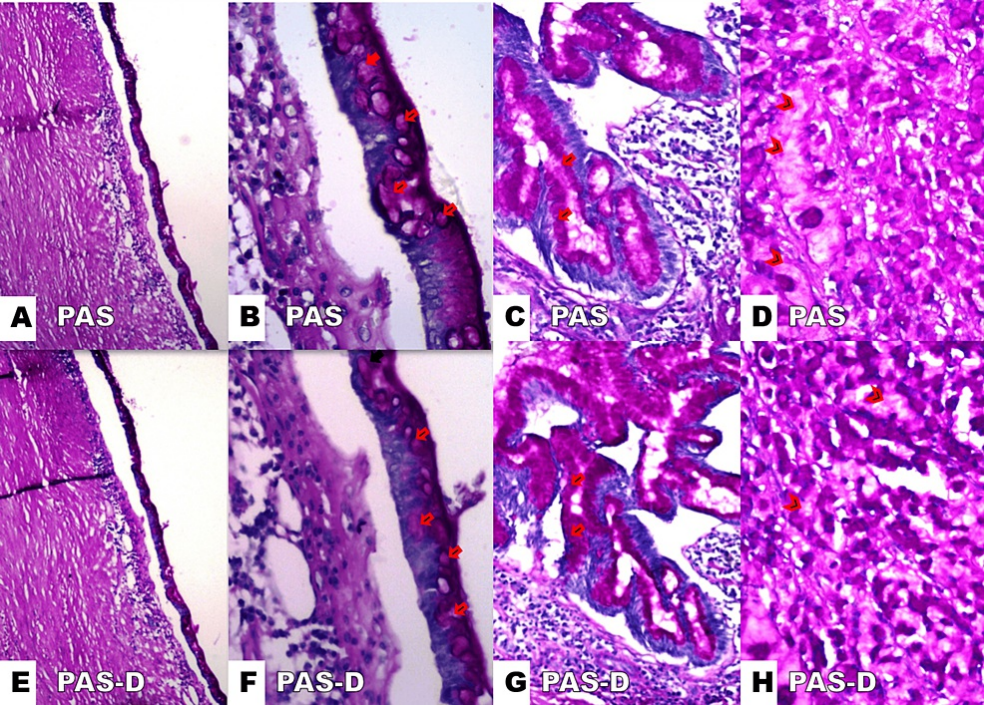
(A-D) Periodic acid Schiff (PAS) and (E-H) PAS-diastase (PAS-D) panels showing magenta color mucin intracellularly (red arrows) and extracellularly (red arrowheads). (A, B, E, F) Renal pelvis lined by tall columnar goblet cells. (C and G) Invasive mucinous glands infiltrate the renal stroma. (D and H) Poorly differentiated areas of signet ring cells with floating magenta extracellular mucin. Original magnification. A: E40x. B: F400x. C: H100x. D: H400x.

The PAS positive staining reaction produces a specific magenta color when reacting with unsubstituted polysaccharides, neutral mucopolysaccharides, mucoproteins, glycoproteins, glycolipids, and phospholipids [5]. Diastase, also known as a-amylase, is an enzyme that selectively breaks down glycogen and is used as an accessory reagent in PAS staining. The process of glycogen removal from tissue produces a negative PAS staining of glycogen so that any remaining PAS staining after diastase treatment must be due to non-glycogen protein-bound neutral polysaccharides, such as mucin [6]. In our case, the PAS reaction was positive, showing magenta-colored diastase-resistant mucin in both extracellular and intracellular spaces (Figure 3).

CK7/CK20

**Figure 4:**
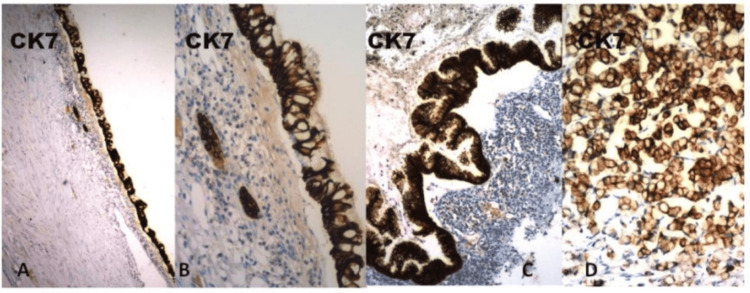
CK7 immunostaining in metaplastic-dysplastic and carcinoma sequence. (A) Transitional and (B) metaplastic epithelium showed diffuse and strong positive CK7 immunostaining. (C) Mucinous adenocarcinoma and signet ring cell component displayed strong cytoplasmic immunoreactivity for CK7. Original magnification A x100; C x200; B,D x400.

**Figure 5:**
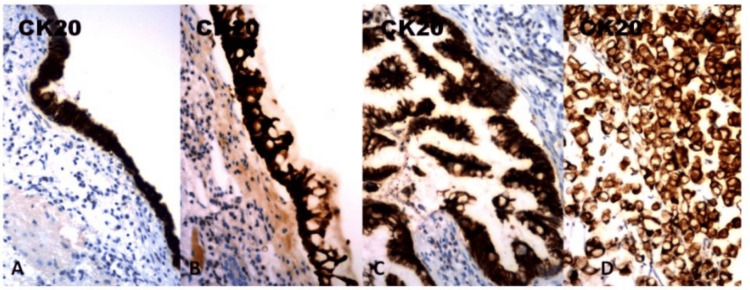
CK20 immunostaining in metaplastic-dysplastic and carcinoma sequence. (A) Transitional and (B) metaplastic epithelium showed diffuse and strong positive CK20 immunostaining. (C) Mucinous adenocarcinoma and the (D) signet ring cell component displayed strong cytoplasmic immunoreactivity for CK20. A: x 100; C: x 200; B, D: x 400.

Immunohistochemistry (IHC) was performed on formalin-fixed, paraffin-embedded tissues using the standard avidin-biotin complex on the automated Ventana Medical Systems utilizing propriety reagents and antibodies [7]. All slides were counterstained with modified Gill’s hematoxylin. Cytokeratins (CKs) represent the epithelial class of intermediate-sized filaments of the cytoskeleton. Comprising 20 subtypes, they have different molecular weights and demonstrate differential expression in various cell types and tumors [6]. Among the most useful CKs are CK7 and CK20. CK7 is found in many ductal and glandular epithelia, including lung, breast, ovary, and endometrium, while CK20 is expressed in the gastrointestinal (GI) epithelium, urothelium, and Merkel cells [8]. The intensity of immunostaining was evaluated semiquantitatively through strong diffuse staining, moderate staining, weak and focal staining, and negative staining. The microsections showed strong and diffuse positive staining for both CK7 and CK20 (Figures 4, 5).

CDX2

**Figure 6:**
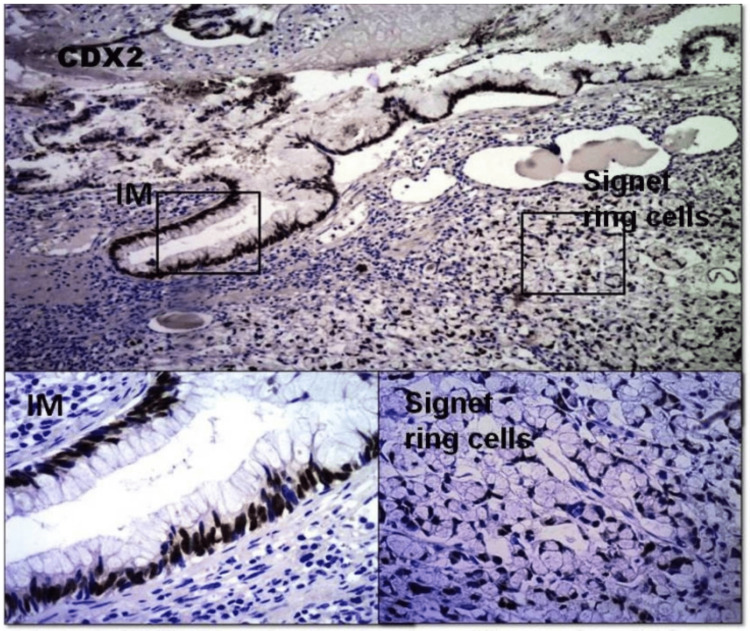
CDX2 expression showing strong diffuse nuclear staining on IM (square) and focal weal staining on clusters of signet ring cells (square). Original magnification 40x, 100x, 100x.

**Figure 7:**
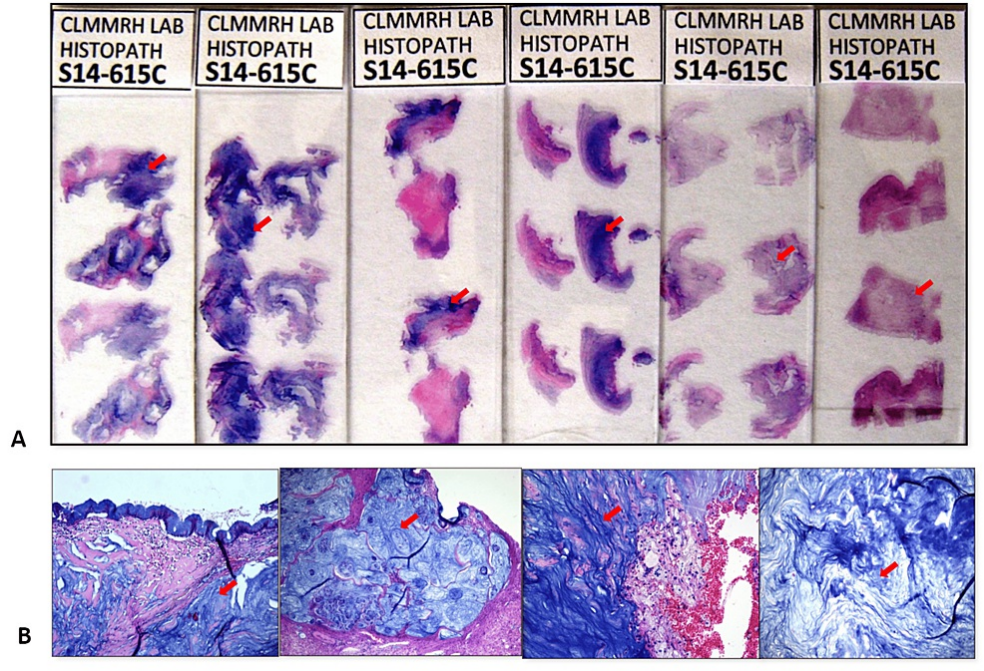
(A) Representative sections showing cyanophilic mucin (arrows). (B) Mucin (arrows) composed 70% of the entire tumor (HE 40x).

CDX2 IHC was performed on formalin-fixed paraffin-embedded tissues using the DAKO Monoclonal Mouse Anti-Human CDX2 antibody (DAKO California, USA) at a 1:50 dilution. The positive cellular staining pattern of CDX2 is typically nuclear. Cytoplasmic staining may be observed in the presence of nuclear staining. Tissue sections from our case showed positive nuclear staining of mucinous carcinoma areas. Strong positive staining was more frequently noted as the metaplasia acquired more dysplastic features. Areas with poorly differentiated signet ring cells showed weak focal positive staining with lower CDX2 staining. The normal adjacent transitional epithelium was negative for CDX2 staining (Figures 6, 7).

## Discussion

The normal urological system is lined by stratified cuboidal epithelium, also known as transitional epithelium or urothelium. It is well recognized that the urothelium can undergo squamous and glandular metaplasia [9]. Metaplasia of the bladder urothelium occurs commonly in response to local injury. Usually, these changes are reversible, but some conditions may be premalignant. In a review of cases, Clouston et al. described that different metaplastic entities are of clinical significance, and keratinizing squamous metaplasia is a precursor to the development of bladder cancer [10]. This is a common event in the urological system. Less commonly experienced is the intestinal metaplasia of the urothelium. Metaplasia of the urothelium typically occurs in reaction to chronic irritation from infection, hydronephrosis, or nephrolithiasis. Dysplasia and carcinoma are postulated to develop from this metaplasia; this is one of the possible carcinogenic sequences in the development of neoplastic mucinous lesions of the renal pelvis [1,2].

Theories proposed for the development of mucinous adenocarcinoma of the urological system include a possible teratomatous origin or coelomic epithelial origin due to its striking resemblance to ovarian mucinous neoplasms. In addition, the propensity of this lesion to arise in anomalous kidneys has led researchers to postulate that these tumors may arise from sequestered renal pelvic epithelium within the parenchyma as a consequence of maldevelopment [11].

Here we described a case of an overtly invasive mucinous adenocarcinoma with signet ring features arising from a background of intestinal metaplasia of the renal pelvis. We attempt to define the intestinal metaplasia-adenocarcinoma sequence of this rare tumor to establish an alternative pathway of carcinogenesis. In this particular case of a 55-year-old male, the kidney transformed into a multiloculated cystic tumor, with a dilated pelvicalyceal system filled with mucin and necrotic debris. The renal outflow tract was obstructed by a 3 × 1.5 × 0.5-cm stone at the renal hilus. Microsections of the tumor revealed a malignant neoplasm disposed in haphazardly arranged glands and epithelial cell clusters invading the desmoplastic stroma or floating in pools of abundant mucin. A minority of the tumor was composed of individually scattered or discohesive clusters of cells with signet ring cell morphology, characterized by abundant intracellular mucin and hyperchromatic, eccentric, crescent-shaped nuclei. Seventy percent of the tumor predominantly consisted of mucin (Figure 7).

The WHO nomenclature for classifying adenocarcinoma of the gastrointestinal tract defines mucinous tumors based on the percentage composition of extracellular mucin (tumors composed of more than 50% mucin). Most mucinous adenocarcinomas contain at least a partial epithelial lining or show free-floating strips of epithelium in the mucin. A variable number of signet ring cells may also be present [12].

The WHO nomenclature for the female genital tract defines mucinous adenocarcinoma as invasive tumors resembling intestinal or endocervical types with ovarian stromal invasion [13]. Mucinous cystadenocarcinoma from non-gastrointestinal and non-ovarian sites is classified differently. For example, a cystic mucinous epithelial neoplasm of the pancreas, which occurs almost exclusively in women, is composed of similar columnar, mucin-producing epithelium but is supported by an ovarian-type stroma [13]. Some pathologists use the female genital tract nomenclature to diagnose mucinous cystadenocarcinoma from non-ovarian sites. In contrast, a case report by Rao et al. expands this diagnostic nomenclature to a case of primary mucinous cystadenocarcinoma involving the renal pelvis in a male patient with an absent ovarian stroma [2]. In that case of a 52-year-old male, the neoplasm demonstrated CK7-positive, CK20-positive, CDX2-negative ovarian-type mucinous cystadenocarcinoma.

With this nomenclature in mind, we propose a case of a de novo intestinal metaplasia carcinogenesis sequence.

We present a case of a tumor that is PAS positive, resulting in the diastase-resistant mucin component of mucinous adenocarcinoma appearing as a bright red color. PAS positive staining reaction produces a specific magenta-color reaction with unsubstituted polysaccharides, neutral mucopolysaccharides, mucoproteins, glycoproteins, glycolipids, and phospholipids [4]. PAS staining with diastase (PAS-D) highlighted the resistance of mucin to digestion by a-amylase. Diastase, or a-amylase, selectively breaks down glycogen. The removal of glycogen from tissue results in a negative PAS staining of glycogen. Therefore, any remaining PAS staining after diastase digestion indicates the presence of non-glycogen, protein-bound neutral polysaccharides, such as mucin [5]. In this study, intracellular mucin and extracellular mucin in the tumor (found within the signet ring cells and intraluminal components) are accented by the bright red color (Figure 3).

The cytokeratin immunohistochemical staining in our case revealed positive CK7 and CK20 staining, showing a strong diffuse cytoplasmic localization in the tumor cells. CK7 is found in many ductal and glandular epithelia, including lung, breast, ovary, and endometrium, but infrequently expressed in colorectal adenocarcinomas, while CK20 is expressed in the gastrointestinal epithelium, urothelium, and Merkel cells [7].

 A study by Bayrak et al. on the CK7/CK20 profiles of intestinal and extraintestinal adenocarcinomas revealed two distinct immunophenotypes: CK7-/CK20+ and CK7+/CK20+ [14]. The CK7-/CK20+ immunophenotype was found in 64% of colorectal and 5% of gastric tumors but not in any pancreatic adenocarcinomas. The CK7+/CK20+ immunophenotype, on the other hand, was observed in 20% of colon, 48% of gastric, and 22% of pancreatic adenocarcinomas, which is the immunophenotype observed in our renal pelvis mucinous adenocarcinoma.

The CK7 reactivity in our case was attributed to the typical expression of CK7 in the urothelium [15]. CK7 reactivity was presumably maintained during metaplastic transformation into an intestinal type and progression into a frank carcinoma. 

CK20 staining in our case was focally positive, as it is typically expressed only in the surface urothelium and benign umbrella cells [16]. With intestinal carcinomatous transformation, CK20 was diffusely and strongly positive, in contrast to normal urothelia.

CDX2 is a 311-amino acid protein with a molecular weight of 33 kDa. It belongs to Class I homeobox genes related to the Drosophila gene caudal, which encodes transcription factors necessary for the development of enteric epithelial cells [17]. This nuclear protein is normally expressed almost exclusively in the intestine, where it plays a role in the proliferation and differentiation of intestinal epithelial cells. Moskaluk et al., using tissue microarrays, showed that strong diffuse CDX2 staining was only observed in the nuclei of normal small and large intestinal epithelium, as well as in portions of the pancreatic duct system [18]. They also demonstrated that colonic adenocarcinomas showed strong extensive staining in 90% of cases. The stomach, esophagus, and ovarian adenocarcinomas showed extensive staining in only 20-30% of cases, with other types of carcinomas showing extensive staining in only 1% or fever cases. Carcinomas from the urothelial tract and kidney tumors do not exhibit CDX2 expression [19].

Representative sections from our case show heterotopic CDX2 positive nuclear staining in areas ranging from intestinal metaplasia to carcinoma. Strong positive staining is more frequently noted as the metaplasia acquire more dysplastic features, with weak focal CDX2 expression in poorly differentiated areas of signet ring cells. This finding is consistent with the research of Lee et al., suggesting that CDX2 expression is associated with intestinal grade and more frequent in the dysplasia group [20]. CDX2 staining is negative in the normal foveolar mucosa of the stomach in Barrett’s metaplasia [18], and it was also negative in the normal transitional epithelium in our case.

In addition to its homeotic function during gut development, CDX2 is also known to play a tumor suppressor role in cancer progression in the distal colon [19]. In our present case, confirming its tumor suppressor role, CDX2 exhibited strong and diffuse expression in IM, dysplasia, carcinoma in situ, and well-differentiated invasive carcinoma, while displaying focal and weak expression in poorly differentiated areas of signet ring cells.

## Conclusions

To the best of our knowledge, this alternative pathway of mucinous tumors involving the renal pelvis has not been fully elucidated in the literature. Pathologists should be aware that the urothelium can undergo metaplastic transformation into a rare subtype. Further investigation of similar tumor subtypes in the urothelial tract should include genome-wide analyses, specifically focusing on KRAS mutation status and microsatellite instability testing, to better understand the molecular basis for the proposed intestinal metaplasia-dysplasia-carcinoma sequence in this rare primary urothelial neoplasm. If this molecular pathogenetic pathway is further defined, therapeutic interventions similar to their intestinal counterparts may be applicable to patients with this type of tumor in the urogenital system.
